# Biological Durability of Wood–Polymer Composites—The Role of Moisture and Aging

**DOI:** 10.3390/ma15238556

**Published:** 2022-12-01

**Authors:** Andreas Buschalsky, Christian Brischke, Kim Christian Klein, Thomas Kilian, Holger Militz

**Affiliations:** 1 Wood Biology and Wood Products, Faculty of Forest Sciences and Forest Ecology, University of Goettingen, Buesgenweg 4, 37077 Goettingen, Germany; 2 Fagus-GreCon Greten GmbH & Co. KG, Hannoversche Straße 58, 31061 Alfeld, Germany; 3 SKZ-KFE gGmbH, Friedrich-Bergius-Ring 22, 97076 Wuerzburg, Germany

**Keywords:** wood–polymer composites, WPC, biological durability, durability test, EN 15534-1, basidiomycetes, soft rot, wood–moisture interaction

## Abstract

Knowledge about the resistance of wood–polymer composites (WPCs) to biological attack is of high importance for purpose-oriented use in outdoor applications. To gain this knowledge, uniform test methods are essential. EN 15534-1 (2018) provides a general framework, including the recommendation of applying a pre-weathering procedure before the biological laboratory tests. However, the procedure’s manner is not specified, and its necessity assumes that a durability test without such pre-weathering will not produce the structural changes that occur during outdoor use. To verify this assumption, this study examined the influence of natural, ground-level pre-weathering on the material properties of different WPC variants, which were tested at intervals of six months in four durability tests under laboratory conditions in accordance with EN 15534-1 (2018). Weathering factors were calculated from determined characteristic values such as mass loss, and loss in moduli of elasticity (MOE) and rupture (MOR). The weathering factors based on mechanical properties tended to decrease with increasing weathering duration. The expected negative influence of pre-weathering on these material properties was thus not confirmed. The weathering factors based on mass loss were subject to high variation. No significant effect of pre-weathering on mass loss due to fungal attack became evident. Overall, the necessity of a pre-weathering step in biological durability tests shall be questioned based on the presented results.

## 1. Introduction

In addition to UV radiation [1], moisture is one of the most important variables influencing the performance, and thus the structural integrity, of wood–polymer composites (WPCs). Due to the hydrophobic character of the polymers predominantly used in WPCs, they absorb very little water and atmospheric moisture. In contrast, the incorporated wood particles are hydrophilic and thus have hygroscopic properties [2,3]. The polysaccharides (cellulose and hemicellulose) in the wood cell walls are responsible for water absorption since they contain numerous accessible OH groups [4,5].

When the first (terrace) decking made of WPCs appeared on the market in the 1990s, it was considered a low-maintenance product with a long service life [6]. The polymer matrix was initially thought to fully encapsulate the wood particles. However, a series of tests showed that the encapsulation was incomplete [7,8], raising questions about the durability of WPCs after a decade of outdoor use. The WPC industry has responded to these problems associated with the first generation of products by improving WPC formulations [9]. Furthermore, manufacturers and distributors made progress in communicating appropriate demands to customers about these products and educating consumers about the proper care and maintenance of WPC products. Although the durability of WPCs continues to be an important issue, a comprehensive understanding of the biological aging of WPC products is pending. Due to the steadily increasing sales of WPC products for outdoor use, especially decking and cladding [10], studies to improve or identify new strategies for protecting WPCs are needed more than ever.

Hitherto, only a few results regarding the biological durability of WPCs are available from outdoor tests. However, knowledge about biological aging processes and their impact on the service performance of WPC products is essential, especially for material and product developers, to counteract this phenomenon during formulation development.

To gain knowledge about the resistance of WPCs to biological attack, uniform test methods are essential. A general normative framework is provided by EN 15534-1 (2018) [11] which, however, only specifies the laboratory test methods for determining resistance to wood-destroying and discoloring organisms (syn.: biological durability). A subsequent recommendation to subject the WPC variants under investigation to an aging procedure before the biological laboratory tests originated from the assumption that durability testing without pre-weathering would not induce the structural changes that occur during outdoor use. In contrast, the biological aging processes of WPC products could be accelerated or initiated by appropriate pretreatment (controlled damage) [12]. However, no specific recommendation for action is given. The goal for the future must therefore be to pursue the question of whether it is at all reasonable to establish a uniform pre-weathering procedure within the standard.

This study attempts to implement a pre-weathering process upstream of the biological laboratory tests, which should correspond as closely as possible to the use scenario of WPCs in outdoor applications. In addition, the need for a pre-weathering procedure is critically assessed.

## 2. Materials and Methods

### 2.1. Selection of Materials

The material selection (Table 1) included various industrially produced WPC profiles (I-1–I-5) containing different polymer matrices (polypropylene (PP), polyethylene (PE), and polyvinyl chloride (PVC)), wood particle contents, and profile variants (hollow or solid profiles). A total of 5 profiles from 4 different manufacturers were examined. Furthermore, WPC variants with known formulations were investigated, compounded at the SKZ—Technology Center Würzburg, and subsequently extruded into profiles (L-1–L-4). The PP-based formulation was varied by the matrix polymer, wood particle type, wood particle content, and the addition of additives.

### 2.2. Mechanical Properties

To evaluate the effects of fungal attack on the mechanical properties of WPCs, the flexural modulus of rupture (MOR) and the flexural modulus of elasticity (MOE) were determined according to EN ISO 178 (2013) [13] on the following test specimens:

1. Untreated WPC test specimens;
2. WPC test specimens after water storage;
3. Reference specimens after air conditioning;
4. WPC and reference test specimens (inoculated and uninoculated) following the durability test against basidiomycetes;
5. WPC and reference test specimens following the durability test against soft rot fungi.

A universal testing machine Z010 TN (Zwick Roell GmbH & Co. KG, Ulm, Germany) was used to determine MOR and MOE. The tests were performed with a preload of 5 N and a test speed of 2 mm min^−1^. The support distance was 64 mm.

### 2.3. Determination of the Material Moisture Content in Different Test Conditions and Conditioning of the Test Specimens

The initial dry mass of untreated WPC specimens (n = 10 of each variant) was determined. After measuring MOR and MOE, the specimens were oven-dried (T = 103 ± 2 °C; t = 48 h) and the initial oven-dry mass was subsequently determined. The water storage of WPC specimens was carried out according to EN 84 (2020) [14]. After 14 days, the test specimens were removed from the impregnation vessel and weighed. Subsequently, MOR and MOE were measured, and the oven-dry mass of 10 replicate specimens after conditioning was determined. Specimens of the reference wood species European beech (*Fagus sylvatica* L.) and Scots pine sapwood (*Pinus sylvestris* L.) were conditioned in a climate chamber (T = 20 ± 2 °C; RH = 65 ± 5%) and weighed. Subsequently, MOR and MOE were measured, and the oven-dry mass of 10 replicas after air conditioning was determined.

### 2.4. Durability Tests

**Figure 1:**
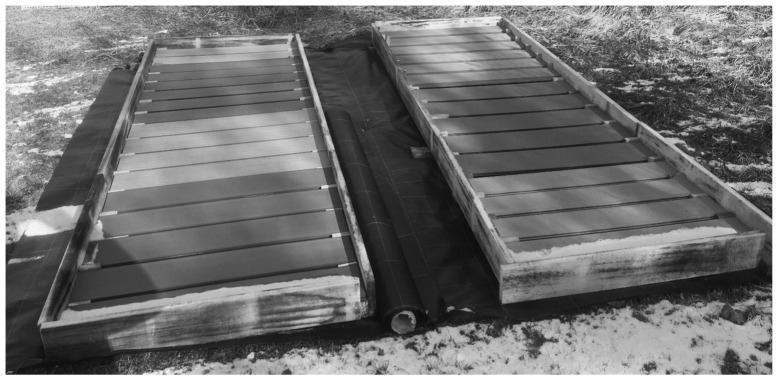
Ground-level pre-weathering of commercial WPC decking in field trials at the North Campus of the University of Goettingen.

A total of four durability tests against basidiomycetes and soft rot fungi in soil were conducted according to EN 15534-1 (2018) using the previously listed industrial and self-manufactured WPC variants. The WPC variants were tested as non-weathered virgin material (0 months) and after natural, ground-level outdoor weathering for 6, 12, and 18 months (Figure 1). For each durability test, non-weathered references were used.

Since not all WPC variants were available at the beginning of the first durability test, the starting times varied (Table 2). Test specimens of 80 (ax.) × 10 × 4 mm³ were made from the various WPCs and the reference wood species. The references served also as virulence control specimens.

#### 2.4.1. Durability Tests against Basidiomycetes

Testing of the durability of the material variants against wood-destroying basidiomycetes was carried out with the two test fungi listed in Table 3. For each WPC and reference variant, 10 replicate specimens were incubated with both test fungi for 16 weeks. Additionally, 10 replicates of WPC and reference specimens were tested without inoculation to gain knowledge about the material moisture of the specimens without a test fungus after completion of the test. Thus, it was possible to determine the extent to which the respective test fungus had an influence on the mechanical properties beyond the influence of moisture during the test.

Kolle flasks prepared with a malt agar were used as test vessels, with each flask containing 2 specimens. To inoculate the Kolle flasks under a clean bench, small round inocula of the test fungi grown in a Petri dish on a malt agar were punched out and placed centrally on the nutrient medium within the Kolle flasks using forceps. Subsequently, the inoculated flasks were stored in a climate chamber (T = 20 ± 2 °C; RH = 65 ± 5%; t = 168 h). Uninoculated Kolle flasks for the moisture control specimens were stored under the same conditions.

Previously conditioned and sterilized specimens of each WPC and reference variant were placed within the Kolle flasks, with 2 specimens in each of them resting on washers. Subsequently, the flasks were again stored in an incubation chamber for 16 weeks (T = 20 ± 2 °C; RH = 65 ± 5%).

At the end of the test period, the specimens were removed from the flasks, cleaned of fungal mycelium, and immediately weighed. Subsequently, MOR and MOE were measured, and the oven-dry mass after completion of the durability test was determined. The latter was used to calculate the mass loss and material moisture content of the specimens.

#### 2.4.2. Durability Tests against Soft Rot Fungi and Other Soil-Inhabiting Microorganisms

Durability against soft rot fungi and other soil-inhabiting microorganisms was tested in so-called terrestrial microcosms (TMCs) using nutrient-rich compost soil. To prepare the test soil, it was sieved with a round sieve (1.2 cm mesh size) and then mixed with sand in a ratio of 70:30. After mixing, the moisture content of the test soil was determined when the water-holding capacity (WHC) was reached. In addition, the amount of water needed to adjust the moisture content to a percentage of 95% of the WHC was calculated in a duplicate determination. Afterward, the soil was transferred into test boxes.

Before starting the test, the WPC specimens were dried (T = 103 ± 2 °C; t = 48 h) and subsequently weighed to obtain the initial oven-dry mass. For each WPC variant, 10 water-stored replicate specimens and the same number of air-conditioned reference specimens were tested. The specimens were inserted vertically into the soil at a distance of 20 mm from each other and from the sides of the test boxes, with an insertion depth of 60 mm. To maintain the substrate moisture, the loaded boxes without lids were weighed and their initial mass was determined before starting the test. At 4-week intervals, the boxes were reweighed and the loss of mass due to water release was compensated by adding water.

After the test period of 16 weeks, the specimens were removed from the test boxes, cleaned of adhering soil, and weighed. Subsequently, MOR and MOE were measured and the oven-dry mass after completion of the durability test was determined.

### 2.5. Influence of Natural Pre-Weathering on the Biological Durability of WPC in Laboratory Tests

To verify the assumption that a natural pre-weathering process reduces the biological durability of WPCs, the characteristic values ‘loss of MOR’, ‘loss of MOE’, and ‘mass loss’, determined before and after the different prestresses and durability tests (Formulas (1)–(3)), were factorized (Formulas (4)–(6)) and plotted against the pre-weathering time.

The relative loss of MOR ($\sigma_{f\;}$) was determined by using Formula (1):

(1) $$\sigma_{f\;}\left(\%\right)=\frac{\sigma_{i}-\sigma_{t}}{\sigma_{i}}$$

$\sigma_{i\;}$→The original MOR of the test specimen of the untreated virgin material (N mm^−2^).
$\sigma_{t\;}$→The MOR of the test specimen of the pre-weathered material after incubation (N mm^−2^).

The relative loss of MOE ($E_{f\;}$) was determined using Formula (2):

(2) $$E_{f\;}\left(\%\right)=\frac{E_{i}-E_{t}}{E_{i}}$$

$E_{i\;}$→The original MOE of the test specimen of the untreated virgin material (N mm^−2^).
$E_{t\;}$→The MOE of the test specimen of the pre-weathered material after incubation (N mm^−2^).

The mass loss ($M_{l\;}$) was determined using Formula (3):

(3) $$M_{l\;}\left(\%\right)=\frac{M_{bI}-M_{aI}}{M_{bI}}$$

$m_{bI\;}$→The dry mass before incubation (g).
$m_{aI\;}$→The dry mass after incubation (g).

The weathering factor for mass loss ($k_{M_{l,\;weathering}\;}$) was determined using Formula (4):

(4) $$k_{M_{l,\;weathering}\;}=\frac{M_{l,\;weathered}}{M_{l,unweathered}}$$

$M_{l,\;weathered}$→The mass loss due to fungal degradation and conditioning after pre-weathering, respectively (%).
$M_{l,\;unweathered}$→The mass loss due to fungal degradation and conditioning without pre-weathering, respectively (%).

The weathering factor for the MOR ($k_{\sigma_{f\;},\;weathering}$) was determined using Formula (5):

(5) $$k_{\sigma f,\mathrm{weathering}}=\frac{\sigma_{f\mathrm{weathered}}}{\sigma_{f\mathrm{unweathered}}}=\frac{\frac{\sigma_{f\mathrm{weathered},\mathrm{untested}}\;-\;\sigma_{f\mathrm{weathered},\mathrm{tested}}}{\sigma_{f\mathrm{weathered},\mathrm{untested}}}}{\frac{\sigma_{f\mathrm{unweathered},\mathrm{untested}}\;-\;\sigma_{f\mathrm{unweathered},\mathrm{tested}}}{\sigma_{f\mathrm{unweathered},\mathrm{untested}}}}$$

$\sigma_{f\;}{}_{weathered}$→The loss of MOR due to fungal degradation and conditioning after pre-weathering, respectively (%).
$\sigma_{f\;}{}_{unweathered}$→The loss of MOR due to fungal degradation and conditioning without pre-weathering, respectively (%).
$\sigma_{f\;}{}_{\;weathered,\;untested}$→The MOR of weathered test specimens without prior exposure to fungi and conditioning, respectively (%).
$\sigma_{f\;}{}_{\;weathered,\;tested}$→The MOR of weathered test specimens after prior exposure to fungi and conditioning, respectively (%).
$\sigma_{f\;}{}_{\;unweathered,\;untested}$→The MOR of non-weathered test specimens without prior exposure to fungi and conditioning, respectively (%).
$\sigma_{f\;}{}_{\;unweathered,\;tested}$→The MOR of non-weathered test specimens after prior exposure to fungi and conditioning, respectively (%).

The weathering factor for the MOE ($k_{E_{f\;},\;weathering}$) was determined using Formula (6):

(6) $$k_{Ef,\mathrm{weathering}}=\frac{E_{f\mathrm{weathered}}}{E_{f\mathrm{unweathered}}}=\frac{\frac{E_{f\mathrm{weathered},\mathrm{untested}}\;-\;E_{f\mathrm{weathered},\mathrm{tested}}}{E_{f\mathrm{weathered},\mathrm{untested}}}}{\frac{E_{f\mathrm{unweathered},\mathrm{untested}}\;-\;E_{f\mathrm{unweathered},\mathrm{tested}}}{E_{f\mathrm{unweathered},\mathrm{untested}}}}$$

$E_{f\;}{}_{weathered}$→The loss of MOE due to fungal degradation and conditioning after pre-weathering, respectively (%).
$E_{f\;}{}_{unweathered}$→The loss of MOE due to fungal degradation and conditioning without pre-weathering, respectively (%).
$E_{f\;}{}_{\;weathered,\;untested}$→The MOE of weathered test specimens without prior exposure to fungi and conditioning, respectively (%).
$E_{f\;}{}_{\;weathered,\;tested}$→The MOE of weathered test specimens after prior exposure to fungi and conditioning, respectively (%).
$E_{f\;}{}_{\;unweathered,\;untested}$→The MOE of non-weathered test specimens without prior exposure to fungi and conditioning, respectively (%).
$E_{f\;}{}_{\;unweathered,\;tested}$→The MOE of non-weathered test specimens after prior exposure to fungi and conditioning, respectively (%).

## 3. Results and Discussion

### 3.1. Mass Loss by Fungal Attack

**Figure 2:**
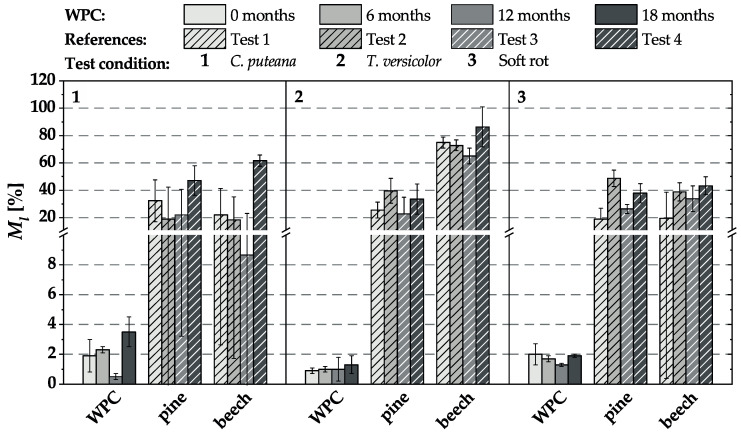
Mass loss (%) of WPC variant I-1 in durability tests without, and after different intervals of, pre-weathering. Only the WPC materials were weathered, not the corresponding references.

The mass losses determined after 16 weeks of incubation (based on the total mass of the test specimens) in the various biological tests and after the different pre-weathering intervals are exemplarily shown in Figure 2 for the WPC variant I-1. *C. puteana* caused an average mass loss between 0.5 and 3.5% and *T. versicolor* between 0.3 and 1.3%. Exposure to unsterile soil led to an average mass loss of less than 2%. The mass loss of the references varied considerably between the four tests, but this was mainly due to the varying virulence of the test fungi. In particular, *T. versicolor* caused very high mass losses in beech, ranging from an average of 65.1 to 74.9%. Overall, *C. puteana* caused lower mass losses in beech, which also varied between 8.7 and 61.7% on average. Mass losses caused by soft rot also varied between tests but were above the required minimum mass loss of 20%, except for the first fungal test (‘0 months’).

In summary, the high fluctuations in mass loss were particularly noticeable in the WPC material and the beech references tested with brown rot, respectively. Furthermore, the very high mass loss of beech due to white rot was noticeable. In addition, the above-described observations of the WPC variant I-1 were confirmed for all other materials. To work around the problem of high mass loss fluctuations between the test intervals and thus to become independent of the respective virulence of the soil substrate, the results of all WPC variants were factorized (Section 3.4) and related to the mass, MOR, and MOE losses of the non-weathered material in the respective durability test.

### 3.2. Loss of MOR and MOE in Durability Tests

**Figure 3:**
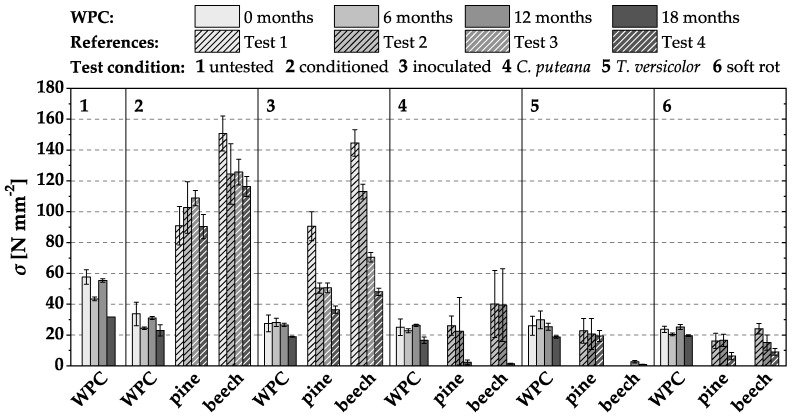
Modulus of rupture (MOR) (N mm^2^) of WPC variant I-1 in durability tests without and after different intervals of pre-weathering. Only the WPC materials were weathered, not the associated references.

**Figure 4:**
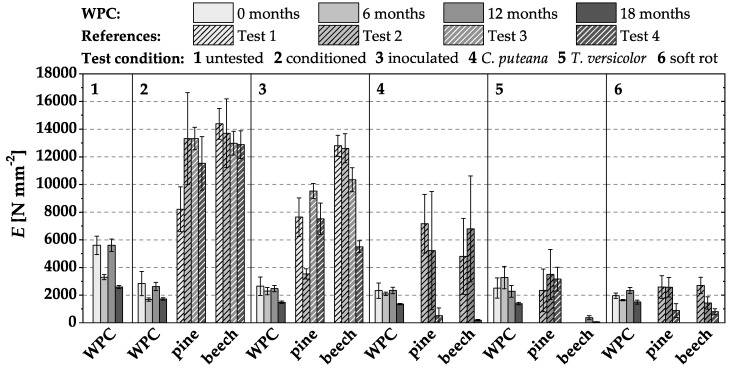
Modulus of elasticity (MOE) (N mm^2^) of WPC variant I-1 in durability tests without, and after different intervals of, pre-weathering. Only the WPC materials were weathered, not the associated references.

**Figure 5:**
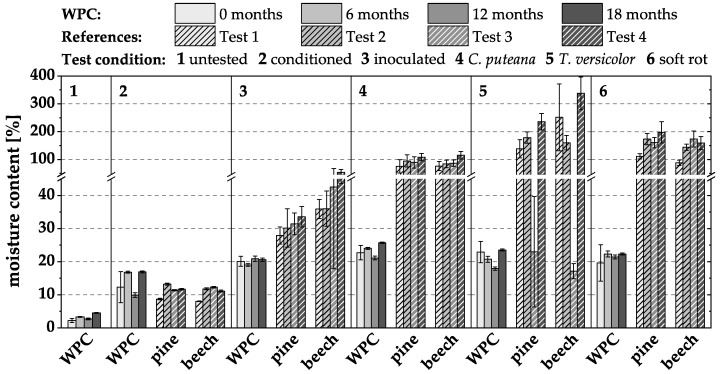
Material moisture content (%) of WPC variant I-1 in durability tests without, and after different intervals of, pre-weathering. Only the WPC materials were weathered, not the associated references.

The MOR and MOE losses determined after 16 weeks of incubation in the different biological tests and after the different pre-weathering intervals are exemplarily shown for the WPC variant I-1 in Figure 3 and Figure 4. On average, the MOR was significantly reduced by water storage by 28 to 55% (Figure 3 ‘conditioned’, i.e., after water storage). Moisture and incubation-induced reductions in MOR were generally only marginally higher (by 27 to 64% on average) than moisture-induced reductions, confirming recently published results by Krause et al. [15]. In contrast, the MOR of the references was significantly more reduced by incubation with fungi than the MOR of the WPC variant I-1 (Figure 3). It was also noticeable that storage of the reference specimens on malt agar only (‘no fungus’) resulted in a decrease in MOR compared to the pre-conditioned specimens. This can be explained by the increase in moisture and the reaching of the fiber saturation point (FSP) within the Kolle flasks (Figure 5), since the moisture content of wood has a high influence on its mechanical properties [16]. Changes in moisture content below FSP have a high influence on MOR and MOE. More precisely, with increasing moisture content up to FSP, MOR and MOE are reduced by about 50% [17]. As the moisture content increases from about 10% (pre-conditioned in a climate chamber) to 30–35% (pine) and 40-50% (beech) inside the Kolle flasks, respectively, FSP is reached or exceeded in the case of the beech references.

Similar to the MOR, the MOE of the WPC specimens was reduced more by water storage (‘conditioned’) than by the subsequent fungal degradation of the wood substance in the durability test (Figure 4). The references also experienced a loss of MOE due to water storage and fungal wood degradation.

The following observations made for WPC variant I-1 were confirmed for all other tested WPC materials:

1. Increased moisture after water storage affected MOR and MOE considerably more than fungal attacks. This finding is in line with previous studies [15,18,19,20,21].
2. Natural pre-weathering of the test specimens did not result in an increased reduction of MOR and MOE.

### 3.3. Material Moisture in Durability Tests

The material moisture contents determined after 16 weeks of incubation in the different biological tests and after the different pre-weathering intervals are exemplarily shown in Figure 5 for the WPC variant I-1. The material moisture content of I-1 was below 5% in the untested condition, and the corresponding wood moisture content was thus below 10%. After water storage, the material moisture content of the WPC averaged between 10 and 17% (related wood moisture content between 14 and 28%). In comparison, the wood moisture content of the references conditioned in normal climate (20 °C/65% RH) averaged between 8 and 12%. Neither incubation with brown and white rot nor exposure in the TMCs led to the considerably higher moisture content of the WPC material compared with the specimens stored in the Kolle flasks without test fungi.

The moisture content of the wood references was considerably higher compared to the WPCs. Beech ‘without fungus’ reached about 50% wood moisture content. After incubation with fungi, its moisture content was clearly above 100%. The wood moisture content of pine ‘no fungus’ was about 27–33%; after incubation, similar to beech, it was well above 100%.

### 3.4. Influence of Natural Pre-Weathering on the Biological Durability of WPC in Laboratory Tests

**Figure 6:**
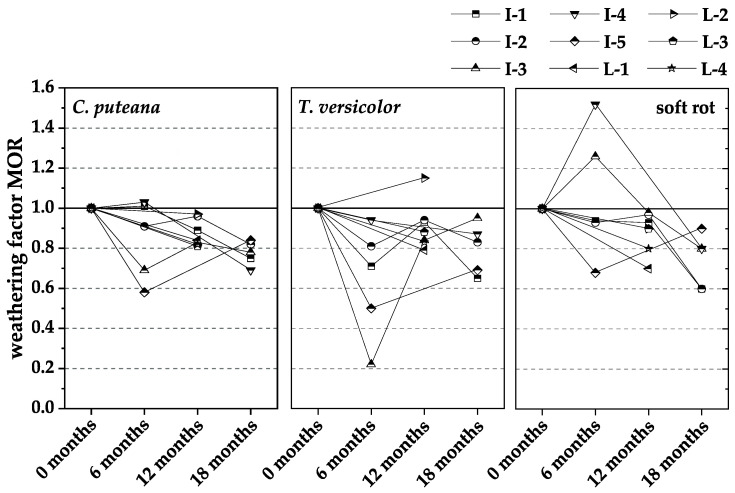
The ratio of loss modulus of rupture (MOR) caused by incubation with *C. puteana*, *T. versicolor*, and soft rot fungi of WPC materials naturally pre-weathered for different intervals to non-weathered WPC references (weathering factors).

**Figure 7:**
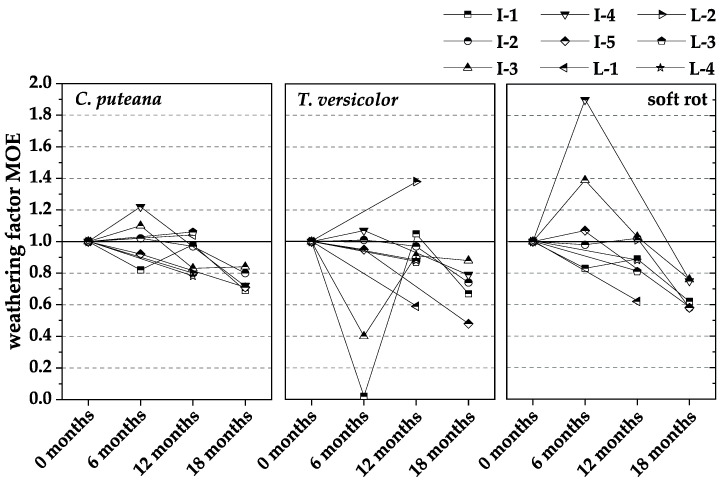
The ratio of loss of modulus of elasticity (MOE) caused by incubation with *C. puteana*, *T. versicolor*, and soft rot fungi of naturally pre-weathered WPC materials of different intervals to non-weathered WPC references (weathering factors).

**Figure 8:**
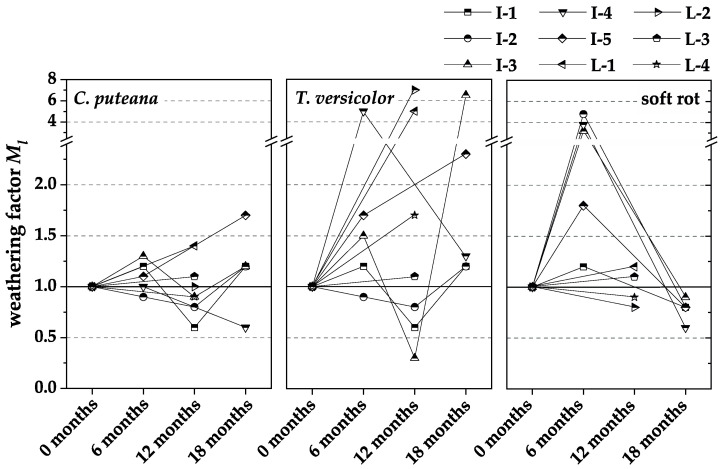
The ratio of mass loss caused by incubation with *C. puteana*, *T. versicolor*, and soft rot fungi of WPC materials naturally pre-weathered for different intervals to non-weathered WPC references (weathering factors).

Figure 6, Figure 7 and Figure 8 show the normalized results of the experienced loss of MOR (Figure 6), MOE (Figure 7), and mass (Figure 8), based on those in the respective durability test. In each case, the losses were related to the non-weathered material. The weathering factors derived from the normalized results were used to validate whether pre-weathering reduces the durability of WPC. The weathering factor in each case is the quotient of the loss due to fungal degradation after weathering divided by the loss due to fungal degradation without weathering. After weathering means the WPC material of the respective test interval, i.e., 6, 12, or 18 months. Without weathering means the WPC material was a new product, which was additionally tested at each test interval. An increase in the factor means that there is a negative influence of pre-weathering on durability, whereas a decrease in the factor means that there is no negative influence of pre-weathering on durability.

The weathering factors for the loss of MOR tended to decrease with increasing weathering duration for all three rot types; thus, the expected negative influence of pre-weathering on the durability of the WPC material was not confirmed. In contrast, the decreasing factors indicated that the pre-exposure of the material to natural weathering reduced the loss of MOR caused by fungal attack over time during the total exposure period of 18 months.

Similar to MOR, the weathering factors for the MOE decreased with increasing weathering duration. However, as described in Section 3.2, the moisture behavior of the WPC materials was not considerably altered by outdoor weathering, so increased moisture is unlikely to cause reduced MOR and MOE values.

The weathering factors for the mass loss were subject to a high variation among each other and over the weathering period. Thus, no apparent effect of pre-weathering on mass loss due to fungal attack could be determined.

## 4. Conclusions

The need for a pre-weathering procedure preceding the biological laboratory tests assumes that a durability test without such pre-weathering will not produce the structural changes that occur during use. In contrast, the calculated weathering factors for the mechanical properties (MOR and MOE) tended to decrease with increasing weathering duration. The expected negative influence of pre-weathering on these material properties was thus not confirmed. The weathering factors for mass loss were subject to high variation among themselves and over the weathering period (especially for the test fungus *T. versicolor*), so no apparent effect of pre-weathering on mass loss due to fungal attack could be determined. Overall, the necessity of pre-stressing the WPC through weathering before biological durability tests shall be questioned based on the presented results.

Furthermore, the results showed in some instances that the considerable loss of elasto-mechanical properties was mainly caused by moisture, and hardly by fungal attack.

## Figures and Tables

**Table 1:** Overview of commercial and self-manufactured WPC variants for testing according to EN 15534-1 (2018).

Label	Specimen ID	Formulation (wt%)		
		Wood	Polymer	Additive
Industry 1	I-1	60	40 (PP) *	n.a.
Industry 2	I-2	50	50 (PVC) *	n.a.
Industry 3	I-3	75	25 (PE) *	n.a.
Industry 4	I-4	75	25 (PE) *	n.a.
Industry 5	I- 5	50	50 (PP) *	n.a.
PP w/softwood	L-1	60	33 (PP)	7 (a + b + c)
PE w/softwood	L-2	60	33.5 (PP)	6.5 (a + b + c)
PP w/hardwood	L-3	60	33 (PP)	7 (a + b + c)
PP w/higher softwood content	L-4	70	23 (PP)	7 (a + b + c)

* The polymer content also includes possible additives: a coupling agent; b stabilizer; c color pigment.

**Table 2:** Overview of WPC variants for testing durability against wood-destroying fungi, including the respective starting times.

Specimen ID	Test 1	Test 2	Test 3	Test 4
I-1	0 months	6 months *	12 months	18 months
I-2	0 months	6 months *	12 months	18 months
I-3	0 months	6 months *	12 months	18 months
I-4	0 months	6 months *		12 months
I- 5	0 months	6 months *	12 months *	18 months
L-1		0 months	6 months *	12 months
L-2		0 months	6 months *	12 months
L-3		0 months	6 months *	12 months
L-4		0 months	6 months *	12 months

* No matched virgin material was tested.

**Table 3:** Test fungi used for durability tests.

Test Fungi	Strain No.	Rot Type
*Coniophora puteana (C. puteana)*	BAM Ebw. 15	Brown rot
*Trametes versicolor (T. versicolor)*	CTB 863 A	White rot
